# Matrix decomposition in meta‐analysis for extraction of adverse event pattern and patient‐level safety profile

**DOI:** 10.1002/pst.2109

**Published:** 2021-03-05

**Authors:** Kentaro Matsuura, Jun Tsuchida, Shuji Ando, Takashi Sozu

**Affiliations:** ^1^ Department of Management Science, Graduate School of Engineering Tokyo University of Science Tokyo Japan; ^2^ Department of Information and Computer Technology, Faculty of Engineering Tokyo University of Science Tokyo Japan

**Keywords:** clinical trial, co‐occurrence, nonnegative matrix factorization, normal dynamic linear model, pattern extraction

## Abstract

The purpose of assessing adverse events (AEs) in clinical studies is to evaluate what AE patterns are likely to occur during treatment. In contrast, it is difficult to specify which of these patterns occurs in each patient. To tackle this challenging issue, we constructed a new statistical model including nonnegative matrix factorization by incorporating background knowledge of AE‐specific structures such as severity and drug mechanism of action. The model uses a meta‐analysis framework for integrating data from multiple clinical studies because insufficient information is derived from a single trial. We demonstrated the proposed method by applying it to real data consisting of three Phase III studies, two mechanisms of action, five anticancer treatments, 3317 patients, 848 AE types, and 99,546 AEs. The extracted typical treatment‐specific AE patterns coincided with medical knowledge. We also demonstrated patient‐level safety profiles using the data of AEs that were observed by the end of the second cycle.

## 1 INTRODUCTION

In order to use medications properly, it is imperative to assess the adverse events (AEs). This assessment is made concomitantly with efficacy evaluations in clinical studies. The purpose of assessing AEs in clinical studies is to evaluate the AE patterns that are likely to occur during treatment. The occurrences of AEs are summarized per treatment, and a meta‐analysis is often applied to the summarized AE data.^1^, ^2^, ^3^, ^4^ However, such a population‐level method does not predict the occurrences of AEs at the patient level, and thus the meta‐analysis cannot be used to determine a patient‐level treatment strategy. At the patient‐level, a predictive model including Poisson‐ or logistic regression uses the occurrence of an AE as an outcome and data such as age, weight, and biomarkers as predictors.^5^, ^6^ In general, however, hundreds of different AE types are observed in clinical trials, with low occurrence frequency. It is impractical to predict all AE types, even though the data of patients characteristics are available. At the patient level, an AE pattern that is likely to occur is sufficient information to support the treatment strategy, even though the occurrence of each AE type cannot be predicted. Therefore, it is important to know which AE pattern occurs at the patient level. We refer to the extent to which a patient has each pattern component as “patient‐level safety profile”.

To obtain AE patterns and patient‐level profiles, this paper utilizes co‐occurrence relationships of patient‐level AEs. For example, among the AEs of anticancer treatments, “vomiting” and “nausea” tend to occur simultaneously. Statistical models dealing with such co‐occurrences include matrix decomposition which could probably extract the “vomiting and nausea pattern.” In other fields, such as natural language processing, matrix decomposition with nonnegative constraints on the elements (nonnegative matrix factorization, NMF^7^, ^8^) is often applied to nonnegative data such as the number of occurrences. For example, one study applied NMF to the number of occurrences of words in web postings or product labels for drugs and was able to extract patterns.^9^ To the best of our knowledge, however, NMF has not yet been applied to patient‐level AEs. Meanwhile, a previous study applied factor analysis, a form of matrix decomposition, to data from approximately 200 patients in a single clinical study and focused on fewer than 20 AEs of interest. Due to the small amount of data, however, only two patterns were extracted and were not used for construction of the patient‐level safety profiles.^10^ Moreover, factor analysis contains strong constraints on the factor score matrix which can cause several issues. For example, the estimation results contain negative values, making the interpretation of patterns and safety profiles difficult.

The aim of this paper is to develop a method which identifies treatment‐specific AE patterns and constructs patient‐level safety profiles. We used patient AE information gathered from treatment onset to a predetermined time point. In this way, we could know the profiles and predict which AEs were likely to occur from the aforementioned time point to the end of treatment.

To this end, we intended to apply NMF to the AE data. However, this could not be realized without developing a new statistical model expanding NMF. Because AEs in clinical studies include both disease‐specific and treatment‐specific events, we needed to build a model to separate them. Moreover, AE data from clinical trials includes severity (ordinal categorical data) and the information of AEs varies with severity; hence, our model was constructed to avoid losing this information. Also, because a single clinical study does not contain sufficient data for our desired analysis, we included the assumption that treatment‐specific AE patterns are similar for drugs with the same mechanism of action. This allowed us to use a sufficient number of patients from multiple clinical studies within a meta‐analysis framework. These expansions enabled us to use actual data to extract AE patterns and demonstrate patient‐level safety profiles.

In Section 2, an example of patient‐level AEs data handled in this paper, our statistical model, and patient‐level safety profile are described. In Section 3, the results from application of these methods are presented. In Section 4, we summarize and discuss our findings.

## 2 DATA AND ANALYSIS METHODS

Although our method was widely applicable to clinical study data with patient‐level AEs, specific data was explained earlier to understand the proposed model in Section 2.2.

### 2.1 Data

Three datasets (registration numbers 118, 120, and 127) were chosen from the Project Data Sphere, which contains patient‐level AE information. Each dataset corresponds to one Phase III study and has only control arm data. These datasets were selected because they were derived from three studies with the highest number of participants. They consisted of breast cancer patients who had undergone chemotherapy. The dosages and disease conditions slightly differed among the studies. Nevertheless, these discrepancies were not considered because we assumed that they had negligible influences on the analysis.

The datasets included AEs that occurred during baseline or treatment periods. Their severities were defined by the Common Terminology Criteria for Adverse Events (CTCAE, 1: mild, 2: moderate, 3: severe, 4: life‐threatening, and 5: death) and ranged from 1 to 4. The baseline data represent AEs occurring during the pre‐treatment period (from registration to randomization). This type of data is generally recorded. In this paper, because alopecia occurred in more than 70% of the patients, its pattern was clear and it was excluded from the analysis. Patients without AEs were also excluded, because their safety profiles were estimated to be zero and their data did not contribute to the estimation of AE patterns. There were 3317 patients and 99,546 AEs analyzed.

The outline of each arm included in the datasets is shown in Table 1. The index *p* is defined for the treatment periods to divide AEs by treatment. For example, a clinical study with *s* = 1 includes one arm named “AC → T,” in which 1625 patients were enrolled and treated with four cycles of AC (doxorubicin + cyclophosphamide) followed by four cycles of T (docetaxel). *p* = 1 represents the baseline period, and the AEs occurring during this period are stored in the matrix ***Y***_1_ as described below. *p* = 2 represents the treatment period using AC, and the AEs that occurred during this period are stored in ***Y***_2_. Similarly, *p* = 3 represents the treatment period using T. The number of patients decreases to 1583 in *p* = 3 because 42 patients dropped out of the study. We also define the treatment index and the mechanism index to facilitate the description of equations. The index of the periods was *p* = 1, …, 10, the index of the studies was *s* ∈ {1,2,3}, the index of the treatments was *t* ∈ {1, …, 5} and the index of the mechanisms of action was *m* ∈ {1, 2}. The study and the treatment were determined by period *p* and the mechanism of action was determined by treatment *t*. Therefore, they were suffixed such as *s*_*p*_, *t*_*p*_, and $m_{t_{p}}$ when they were accessed using *p*. For example, *s*_3_ = 1, *t*_3_ = 5 and *m*_5_ = 2.

**TABLE 1:** Summary of each arm and indices in the three datasets

Study index *s*	Arm	Period index *p*	Treatment	Treatment index *m*	Mechanism index *t*	Number of patients	Number of cycles
1	AC→T	1	Baseline	NA	NA	1625	NA
1	AC→T	2	AC	1	1	1625	4
2	AC→T	3	T	5	2	1583	4
2	FAC	4	FAC	2	1	731	6
3	A→CMF	5	Baseline	NA	NA	480	NA
3	A→CMF	6	A	3	1	480	4
3	A→CMF	7	CMF	4	1	459	3
3	AC→CMF	8	Baseline	NA	NA	481	NA
3	AC→CMF	9	AC	1	1	481	4
3	AC→CMF	10	CMF	4	1	472	3

*Note: s* represents the index of the studies and *p* represents the index of the periods. A is doxorubicin, C is cyclophosphamide, F is 5‐fluorouracil, M is methotrexate, and T is docetaxel. AC, FAC, and CMF represents combination therapies. *t* represents the index of the treatments and *m* represents the index of the mechanisms. Docetaxel is a taxane‐based anticancer drug that binds to microtubules and inhibits mitosis. The other drugs inhibit DNA or RNA synthesis and are often used in combination. Their AEs are relatively similar. Therefore, two mechanisms of action were assumed here, namely, inhibition of nucleic acid synthesis and inhibition of mitosis. Cycles represent the lengths of the treatment periods.

In this paper, AEs with the same name but different severities were distinguished. The combination of name and severity was treated as “AE type”. For example, VOMIT with severity 2 is “VOMIT_2.” Consequently, the total number of AE types was *J* = 848.

These datasets included the number of occurrences of each AE type per cycle. The number of occurrences was summarized per period *p* and the result was expressed as *N*_*p*_ × *J* matrix ***Y***_*p*_ (*p* = 1, …, 10), where *N*_*p*_ is the number of patients who participated in period *p*. ***Y***_*p*_ was a sparse matrix in which many elements were 0. $Y_{\mathrm{ij}}^{\left(p\right)}$, which is the (*i*, *j*) element of ***Y***_*p*_, represents the number of occurrences of AE type *j* for patient *i* during period *p*.

### 2.2 Analysis methods

#### 2.2.1 Statistical model

Here, the statistical model for generating ***Y***_*p*_ (*p* = 1, …, 10) is explained. Scalars are in italics, vectors are in bold font, and matrices are in bold italics.

Hereafter, one period *p* was selected and fixed. ***Y***_*p*_ was written as ***Y***, *N*_*p*_ as *N*, *s*_*p*_ as *s*, *t*_*p*_ as *t*, and $m_{t_{p}}$ as *m*. The patient index was written as *i* and the AE type index was written as *j*.

Because ***Y*** was count data, it was assumed that each element of ***Y*** independently followed the following Poisson distribution:

(1) $$Y_{\mathrm{ij}}∼\text{Poisson}\left(C_{i}\lambda_{\mathrm{ij}}\right)\;i=1,\ldots ,N\;j=1,\ldots ,J$$

where *C*_*i*_ is a constant representing the length of the treatment period of patient *i* (i.e., the number of cycles in the aforementioned data).

*λ*_*ij*_ was separated into $\beta_{\mathrm{ij}}^{\left(t\right)}$ derived from treatment *t* and $\alpha_{\mathrm{ij}}^{\left(s\right)}$ derived from study *s*. In other words,

(2) $$\lambda_{\mathrm{ij}}=\beta_{\mathrm{ij}}^{\left(t\right)}+\alpha_{\mathrm{ij}}^{\left(s\right)}\;i=1,\ldots ,N\;j=1,\ldots ,J$$

where $\alpha_{\mathrm{ij}}^{\left(s\right)}$ is a parameter per study representing the occurrence intensity of each AE caused by disease, radiotherapy, etc. For the baseline data, only $\alpha_{\mathrm{ij}}^{\left(s\right)}$ was considered and it was assumed that $\beta_{\mathrm{ij}}^{\left(t\right)}=0$.

$\beta_{\mathrm{ij}}^{\left(t\right)}$ is the (*i*, *j*) element of the *N* × *J* matrix ***β*** ^(*t*)^, and $\alpha_{\mathrm{ij}}^{\left(s\right)}$ is the (*i*, *j*) element of the *N* × *J* matrix ***α*** ^(*s*)^. The occurrence patterns of AEs are usually limited.^11^ Therefore, it was assumed that they consisted of a combination of relatively few patterns with little information loss and the matrix decomposition was applied as follows:

(3) $$\beta^{\left(t\right)}=\theta^{\left(t\right)}\phi^{\left(t\right)}$$

(4) $$\alpha^{\left(s\right)}=\xi^{\left(s\right)}\eta^{\left(s\right)}$$

where ***θ*** ^(*t*)^ is an *N* × *K* matrix, ***ϕ*** ^(*t*)^ is a *K* × *J* matrix, ***ξ*** ^(*s*)^ is an *N* × *L* matrix, and ***η*** ^(*s*)^ is an *L* × *J* matrix. *K* and *L* are ≪*J* and represent the numbers of AE type occurrence patterns. ***ϕ*** and ***η*** are common to all patients and represent the occurrence intensities of all AE types determined for each pattern. ***θ*** and ***ξ*** represent the extent to which each patient has each pattern component. Expressing a matrix by the product of low‐rank matrices generally facilitates interpretation and improves prediction accuracy by eliminating noise.

The decomposition of ***β*** into ***θ*** and ***ϕ*** is not unique. The constraints for ***θ*** and ***ϕ*** make them identifiable except the order of the components and make the estimation possible. For example, the constraint that ***θ*** and ***ϕ*** are orthogonal matrices makes matrix decomposition equivalent to principal component analysis. However, the approximation of ***Y*** is poor because orthogonality is a strong constraint. It is difficult to interpret the result because certain elements of ***θ*** and ***ϕ*** are negative. Moreover, some *λ*_*ij*_, which represents the average number of occurrences, may also be negative. Therefore, in this paper, the constraint that all elements of ***θ*** and ***ϕ*** are nonnegative was added (NMF).

***θ***^(*t*)^ was reparametrized as follows:

(5) $$\theta^{\left(t\right)}=\exp{\tilde{\theta }}^{\left(t\right)}$$

where ${\tilde{\theta }}^{\left(t\right)}$ is an *N* × *K* matrix, and the exp function operates on each element and returns a matrix.

For ***ϕ*** ^(*t*)^, background knowledge of treatments was used, and it was assumed that ***ϕ*** ^(*t*)^ is constructed as follows:

(6) $$\Phi_{k}^{\left(t\right)}=\text{softmax}\left(M_{k}^{\left(m\right)}+R_{k}^{\left(t\right)}\right)\;k=1,\ldots ,K$$

where $\Phi_{k}^{\left(t\right)}$ represents the row vector of the *k*‐th row of ***ϕ*** ^(*t*)^. **u** = softmax(**v**) converts vector **v** into vector **u** whose elements are nonnegative and have a sum of 1, using the following formula:

$$u_{j}=\frac{\exp v_{j}}{\sum_{j}\exp v_{j}}$$

Equation (6) facilitates comparisons between treatments because the sum of *J* elements of each row (pattern) of ***ϕ*** ^(*t*)^ becomes 1. ***M*** ^(*m*)^ is a *K* × *J* matrix representing the mean effect of the mechanism of action *m*. ***R*** ^(*t*)^ is a *K* × *J* matrix representing the random effect of treatment *t*. ***M*** ^(1)^ and ***M*** ^(2)^ were considered for all data. For “AC,” “FAC,” “A,” and “CMF,” which inhibit nucleic acid synthesis, the random effects of ***R*** ^(1)^, ***R*** ^(2)^, ***R*** ^(3)^, and ***R*** ^(4)^ were considered, respectively (see Table 1).

Similar constraints were assumed for ***α*** ^(*s*)^ as in ***β*** ^(*t*)^. ***ξ*** ^(*s*)^ was reparametrized as follows to make each element nonnegative:

(7) $$\xi^{\left(s\right)}=\exp{\tilde{\xi }}^{\left(s\right)}$$

***η***^(*s*)^ was defined using *L* × *J* matrix ***S*** ^(*s*)^ as follows:

(8) $$\eta_{l}^{\left(s\right)}=\text{softmax}\left(S_{l}^{\left(s\right)}\right)\;l=1,\ldots ,L$$

If AEs caused by disease were of interest, $S_{l}^{\left(s\right)}$ could be divided into mean‐ and random effects as for the treatments.

In an occurrence pattern, AE types with the same name and similar severities (e.g., NAUSEA_2 and NAUSEA_3) should have similar occurrence intensities. Therefore, ***M*** ^(*m*)^, ***R*** ^(*t*)^ and ***S*** ^(*s*)^ were constrained using a NDLM (normal dynamic linear model),^12^ which is often applied in dose–response curves:

(9) $$M_{kj^{\prime }}^{\left(m\right)}∼N\left(M_{\mathrm{kj}}^{\left(m\right)},\sigma_{G}^{2}\right)\;k=1,\ldots ,K\;\left(j,j^{\prime }\right)\;\in \;J$$

(10) $$R_{kj^{\prime }}^{\left(t\right)}∼N\left(R_{\mathrm{kj}}^{\left(t\right)},\sigma_{G}^{2}\right)\;k=1,\ldots ,K\;\left(j,j^{\prime }\right)\;\in \;J$$

(11) $$S_{lj^{\prime }}^{\left(s\right)}∼N\left(S_{\mathrm{lj}}^{\left(s\right)},\sigma_{G}^{2}\right)\;l=1,\ldots ,L\;\left(j,j^{\prime }\right)\;\in \;J$$

where J is a set of the combination of AE types *j* and *j* ^′^ satisfying the conditions that *j* and *j* ^′^ have the same AE name, their severities are adjacent, and *j* < *j* ^′^.

The addition of a constant to each element of the argument vector does not change the output of the softmax function. Therefore, ***M*** ^(*m*)^, ***R*** ^(*t*)^ and ***S*** ^(*s*)^ were constrained as follows so that they could be identified and estimated:

(12) $$M_{\mathrm{kj}}^{\left(m\right)}∼N\left(0,\sigma_{M}^{2}\right)\;k=1,\ldots ,K\;j\;\in \;J$$

(13) $$R_{\mathrm{kj}}^{\left(t\right)}∼N\left(0,\sigma_{R}^{2}\right)\;k=1,\ldots ,K\;j\;\in \;J$$

(14) $$S_{\mathrm{lj}}^{\left(s\right)}∼N\left(0,\sigma_{S}^{2}\right)\;l=1,\ldots ,L\;j\;\in \;J$$

where J is a set of AE type *j* satisfying the condition that *j* has the lowest severity in each AE name.

Considering Equations (1), (2), (3), (4), (5), (6), (7), (8), (9), (10), (11), (12), (13), (14)) for all data ***Y***_*p*_ (*p* = 1, …, 10), the log of the posterior probability was constructed as follows:

(15) $$\begin{array}{ll} & \log P\left({\tilde{\theta }}^{\left(t\right)},M^{\left(m\right)},R^{\left(t\right)},{\tilde{\xi }}^{\left(s\right)},S^{\left(s\right)}\;|\;Y_{p},\sigma_{M},\sigma_{R},\sigma_{S},\sigma_{G}\right)\\ & =\sum_{p=1}^{10}\sum_{i=1}^{N_{p}}\sum_{j=1}^{J}\log\text{Poisson}\left(Y_{\mathrm{ij}}^{\left(p\right)}\;|\;C_{i}^{\left(p\right)}{\left[\exp{\tilde{\theta }}^{\left(t_{p}\right)}\text{softmax}\left(M^{\left(m_{t_{p}}\right)}+R^{\left(t_{p}\right)}\right)+\exp{\tilde{\xi }}^{\left(s_{p}\right)}\text{softmax}\left(S^{\left(s_{p}\right)}\right)\right]}_{\mathrm{ij}}\right)\\ & +\sum_{m=1}^{2}\sum_{k=1}^{K}\left[\sum_{j\;\in \;J}\log N\left(M_{\mathrm{kj}}^{\left(m\right)}\;|\;0,\sigma_{M}^{2}\right)+\sum_{\left(j,j^{\prime }\right)\;\in \;J}\log N\left(M_{kj^{\prime }}^{\left(m\right)}\;|\;M_{\mathrm{kj}}^{\left(m\right)},\sigma_{G}^{2}\right)\right]\\ & +\sum_{t=1}^{5}\sum_{k=1}^{K}\left[\sum_{j\;\in \;J}\log N\left(R_{\mathrm{kj}}^{\left(t\right)}\;|\;0,\sigma_{R}^{2}\right)+\sum_{\left(j,j^{\prime }\right)\;\in \;J}\log N\left(R_{kj^{\prime }}^{\left(t\right)}\;|\;R_{\mathrm{kj}}^{\left(t\right)},\sigma_{G}^{2}\right)\right]\\ & +\sum_{s=1}^{3}\sum_{l=1}^{L}\left[\sum_{j\;\in \;J}\log N\left(S_{\mathrm{lj}}^{\left(s\right)}\;|\;0,\sigma_{S}^{2}\right)+\sum_{\left(j,j^{\prime }\right)\;\in \;J}\log N\left(S_{lj^{\prime }}^{\left(s\right)}\;|\;S_{\mathrm{lj}}^{\left(s\right)},\sigma_{G}^{2}\right)\right] \end{array}$$

where the softmax function operates on each row of a matrix and returns a matrix, *Poisson*(*y* |  *λ*) represents the probability mass at *y* of the Poisson distribution with parameter *λ*, and $N\left(y\;|\;\mu ,\sigma^{2}\right)$ represents the probability density at *y* of the normal distribution with parameters *μ* and *σ* ^2^. In the Appendix A, we explain that the models and Equation (15) can be simplified under certain conditions.

#### 2.2.2 Estimation

*θ*^(*t*)^, ***M***^(*m*)^, ***R***^(*t*)^, ***ξ***^(*s*)^, and ***S***^(*s*)^ were estimated by maximizing the log‐probability log*P* in Equation (15). *σ*_*G*_, *σ*_*M*_, *σ*_*R*_, and *σ*_*S*_ could also be estimated if there was sufficient data. In this paper, they were regarded as hyperparameters and assigned fixed values. Although the number of parameters is large, the parameters are identifiable because matrix decomposition is an informative constraint,^7^ and Equations (6), ((8), (9), (10), (11), (12), (13), (14)) do not cause the loss of identifiability.

The software used for this estimation were Stan 2.17.^13^ Maximum a posteriori (MAP) estimation was performed using the limited‐memory Broyden‐Fletcher‐Goldfarb‐Shanno (L‐BFGS) algorithm. The Stan and R^14^ programs are available on GitHub,^15^ and statisticians can use our method by modifying parts of the programs.

#### 2.2.3 Hyperparameter determination

The number of patterns *K* and *L* were hyperparameters that had to be fixed before estimation. Although there are several determination methods, the technique applied here ensured hyperparameter interpretation according to background information. If the number of patterns was too small, several were combined and extracted as one pattern. As a rule, however, this manipulation should be avoided. Conversely, if the number of patterns was large, similar patterns were extracted.

Other determinations were based either on predictive performance or information criteria, such as the Akaike Information Criterion (AIC) and the Widely Applicable Information Criterion (WAIC).^16^ On the other hand, one study indicated that results derived from the number of patterns determined by these methods do not align with the actual interpretation.^17^

In this paper, the hyperparameters were determined as follows. The small value *L* = 2 was selected because there are far fewer AEs associated with the disease itself than those originating from anticancer treatments used in the clinical studies. The number of detectable patterns associated with the disease was assumed to be small. In fact, in the data in Section 2.1, less than 10% of the total AEs could be attributed to the disease. *K* = 15 was set because similar patterns occur when *K* is assigned higher values. For actual clinical studies, *L* and *K* must be determined after trying several values and discussing the results.

*σ*_*G*_ = 1.5 because the number of severities was as low as 1 to 4. *σ*_*M*_ = 5 and *σ*_*S*_ = 5 were sufficiently large to express any pattern and *σ*_*R*_ = 0.5 because it was assumed that the magnitude of the random effect was about 10% that of the mean effect.

### 2.3 AE patterns

The estimate of ***ϕ*** ^(*t*)^ is a set of AE type patterns specific to treatment *t*. $\Phi_{k}^{\left(t\right)}$ is pattern *k* and a vector of occurrence intensities of all AE types. AE types most likely to occur in pattern *k* are readily confirmed by rearranging its intensities in descending order and plotting them in a table or graph. Patterns where severe AEs are most likely to occur can also be confirmed.

The estimate of $\theta_{i}^{\left(t\right)}$ represents responsiveness of patient *i* to treatment *t*, and it provides condensed information of **Y**_*i*_ through the patterns. $\theta_{\mathrm{ik}}^{\left(t\right)}$ is the extent to which patient *i* has a pattern *k* component. ***θ*** ^(*t*)^ can also be interpreted as the soft clustering result for the patients, where each patient can have components belonging to multiple clusters. The number of patients with high values of a pattern *k* component can be established by visualizing the distribution of $\theta_{:k}^{\left(t\right)}$, which represents the *k*‐th column of ***θ*** ^(*t*)^ (a column vector).

### 2.4 Patient‐level safety profile

Suppose that patient *i* ^′^ receives treatment *t* and his/her AEs (i.e., $Y_{i^{\prime }}$) are assessed to estimate $\theta_{i^{\prime }}^{\left(t\right)}$ at a particular time point in a new clinical study.

All parameters can be estimated from the data, including the AEs of patient *i* ^′^ obtained from the start of the treatment to the specified time point and the AEs from other patients and other clinical studies. The estimation will reveal which pattern components are abundant in patient *i* ^′^ and predict which AE types are most likely to occur from the specified time point to the end of treatment. This method is effective if computation time and resources suffice. However, it is not realistic to repeat parameter estimation whenever the assessment time point changes.

When prompt predictions are needed during a new clinical study, diverting the already estimated ***ϕ*** ^(*t*)^ (e.g., our estimated ***ϕ*** ^(*t*)^) enables rapid $\theta_{i^{\prime }}^{\left(t\right)}$ estimation. Assuming that ***ϕ*** ^(*t*)^ are sufficiently close to the true values and the influence of each study (i.e., $\alpha_{i^{\prime }j}^{\left(s\right)}$) is sufficiently small, we maximize the following log‐probability to estimate $\theta_{i^{\prime }}^{\left(t\right)}$:

(16) $$\sum_{j=1}^{J}\log\;\text{Poisson}\left(Y_{i^{\prime }j}\;|\;C\left[\sum_{k=1}^{K}\left(\exp{\tilde{\theta }}_{i^{\prime }k}^{\left(t\right)}\right)\phi_{\mathrm{kj}}^{\left(t\right)}\right]\right)$$

where *C* is a constant representing the length of the treatment period of patient *i* ^′^. The posterior distribution may also be estimated by setting noninformative prior distributions or appropriate prior distributions for ${\tilde{\theta }}_{i^{\prime }}^{\left(t\right)}$.

The estimate of $\theta_{i^{\prime }}^{\left(t\right)}$ and Equations (2), (3), (4)) indicate that $\lambda_{i^{\prime }j}$ can be calculated. $\lambda_{i^{\prime }j}$ is the mean number of occurrences of AE type *j* in a future treatment period. AE types with high $\lambda_{i^{\prime }j}$ must be carefully monitored.

## 3 RESULTS

We applied the method described in Section 2.2 to the data in Section 2.1 for AE pattern extraction and patient‐level safety profile estimation. The results corresponding to Sections 2.3 and 2.4 are explained below.

### 3.1 AE patterns

Here, the ***ϕ*** ^(*t*)^ patterns of treatment AC (*t* = 1) are shown. For the patterns of all treatments, see Table S1.

The 15 patterns of ***ϕ*** ^(1)^ were numbered in descending order of the log‐probability increase. The results of patterns 1 to 15 are shown in Table 2. Because the log‐probability increase involving patterns 1 to 8 was greater than 80% of that for all patterns, we focused on the results from patterns 1 to 8.

**TABLE 2:** Result of ***ϕ*** ^(1)^

Pattern *k*	AE type	$\phi_{k,j}^{\left(1\right)}$
1	NAUSEA_1	99.9
2	VOMIT_2	44.7
	NAUSEA_2	42.4
	**VOMIT_3**	**6.2**
	**NAUSEA_3**	**6.0**
3	ASTHENIA_1	99.9
4	CONSTIP_1	53.7
	STOMATITIS_2	23.6
	WEIGHT INC_1	17.6
	WEIGHT INC_2	1.8
	INFECT_2	1.3
	**STOMATITIS_3**	**0.6**
5	VOMIT_1	99.8
6	TASTE PERVERS_1	22.5
	DIARRHEA_1	19.4
	INSOMNIA_1	16.8
	LACRIMATION DIS_1	13.9
	MENS DIS_1	13.2
	SKIN DRY_1	4.7
	FEVER_1	3.3
	PHARYNGITIS_1	1.9
	**STOMATITIS_3**	**0.1**
7	NAUSEA_2	46.1
	NAIL DIS_1	24.6
	VASODILAT_2	13.9
	ANOREXIA_2	6.3
	PAIN ABDO_2	2.7
	NAIL DIS_2	1.7
	WEIGHT DEC_1	1.4
	**ANOREXIA_3**	**0.3**
	**WEIGHT DEC_3**	**0.1**
8	STOMATITIS_1	99.3
	**STOMATITIS_3**	**0.3**
9	ASTHENIA_2	95.6
	**ASTHENIA_3**	**4.1**
10	HEADACHE_1	39.8
	MYALGIA_1	20.7
	RHINITIS_1	19.1
	ARTHRALGIA_1	13.5
	SKIN DIS_1	3.5
	**DIARRHEA_3**	**0.2**
Pattern *k*	AE type	$\phi_{k,j}^{\left(1\right)}$
11	VASODILAT_1	30.6
	ANOREXIA_1	29.9
	PAIN_1	19.5
	CONSTIP_2	13.4
	DEPRESSION_1	2.6
	CONJUNCTIVITIS_1	1.2
	**CONSTIP_3**	**0.2**
12	NEUROPATHY_1	17.6
	DYSPNEA_2	10.3
	RASH MAC PAP_1	9.9
	EDEMA PERIPH_1	8.1
	INSOMNIA_2	7.0
	DIZZINESS_1	6.7
	INFECT_1	4.7
	TASTE PERVERS_2	2.9
	**DYSPNEA_3**	**0.6**
	**HEADACHE_3**	**0.2**
13	**MENS DIS_3**	**10.0**
	PAIN ABDO_1	6.9
	**INFECT_3**	**5.0**
	DIARRHEA_2	4.3
	PAIN BACK_1	3.8
	WEIGHT DEC_1	3.4
	SWEAT_1	3.4
	ARTHRALGIA_2	2.9
14	DYSPEPSIA_1	49.1
	HEADACHE_2	21.2
	DYSPEPSIA_2	15.9
	INJECT SITE REACT_1	8.7
	HEADACHE_1	1.6
	**HEADACHE_3**	**0.5**
	**DYSPEPSIA_3**	**0.4**
15	CONJUNCTIVITIS_1	10.9
	COUGH INC_1	10.1
	ANXIETY_1	9.5
	DRY MOUTH_1	7.5
	PAIN BONE_1	4.7
	DRY EYE_1	4.4
	ALLERG REACT_1	3.9
	PALPITAT_1	3.7
	**BRONCHITIS_3**	**0.3**
	**ALLERG REACT_3**	**0.3**

*Note*: The unit of occurrence intensity was converted to % because the sum of occurrence intensities per pattern was set to 1 by the softmax function. The top eight AE types whose occurrence intensities are more than 1% are presented for each pattern. We also show the top two AE types with severity 3 or higher and whose occurrence intensities are more than 0.1% (bold letters).

For instance, pattern 2 is a “nausea and vomiting pattern” in which nausea and vomiting of severities 2 and 3, respectively, tended to occur simultaneously. However, nausea and vomiting of severity 1 were segregated into patterns 1 and 5 because the numbers of their occurrences were very large. Pattern 4 was interpreted as a “constipation pattern” and included constipation, stomatitis, and weight increase. Pattern 6 was the “neuropathy pattern” involving taste perversion, diarrhea, insomnia, and lacrimation disorder. Pattern 7 was another “neuropathy pattern” with nausea of severity 2, nail disorder, vasodilation, anorexia, and weight decrease.

**FIGURE 1:**
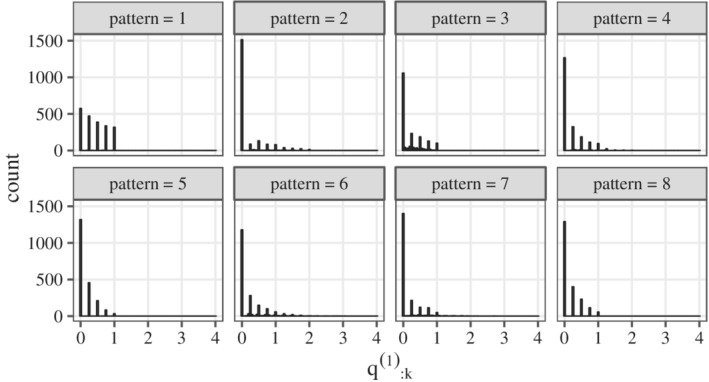
Distributions of $\theta_{:k}^{\left(1\right)}$_._ *Note*: The distribution was partially discrete because the number of occurrences of AEs and the treatment periods (that is, the number of cycles) were integers here

Next, we confirmed the responsiveness of the 2106 patients being administered AC. The distributions of $\theta_{:k}^{\left(1\right)}$ corresponding to patterns 1 to 8 are shown in Figure 1. The $\theta_{:k}^{\left(1\right)}$ for most patients was almost 0 but $\theta_{:k}^{\left(1\right)}$ was high in a few cases.

For the remaining treatments, we can also extract 15 patterns ***ϕ*** ^(*t*)^ and plot distributions of $\theta_{:k}^{\left(1\right)}$.

### 3.2 Patient‐level safety profile

We demonstrated patient‐level safety profiles using the method in Section 2.4. Suppose that four new patients (*i* ^′^ = 1^′^, …, 4^′^) received AC in a new clinical study. Fixing ***ϕ*** ^(1)^ to the values in Section 3.1 and applying Equation (16), we estimated $\theta_{i^{\prime }}^{\left(1\right)}$ by MAP estimation every time an AE occurred. $\theta_{i^{\prime }}^{\left(1\right)}$ can be calculated for any AE data. For example, the AE data of the four patients were assumed to be the same as *i* = 548,338,871,530, who received AC, respectively (see Data S1).

**FIGURE 2:**
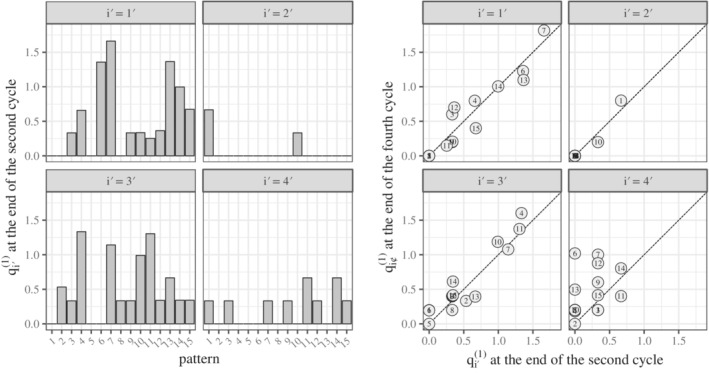
Estimated $\theta_{i^{\prime }}^{\left(1\right)}$ of the four patients

The estimated $\theta_{i^{\prime }}^{\left(1\right)}$ at the end of the second cycle is shown in Figure 2 (left). In patient 1^′^, pattern 7 component (i.e., $\theta_{1^{\prime }7}^{\left(1\right)}$) was very high due to vasodilation of severity 2 and abdominal pain of severity 2. Abdominal pain of severity 2 was a rare AE type that occurred in only 1.9% (40 out of 2106) of patients who received treatment AC, contributing to an increase in $\theta_{1^{\prime }7}^{\left(1\right)}$ because it is a pattern‐7‐specific AE type. Patient 2^′^ had only two pattern components. The pattern components of patient 3^′^ were separated into high and low. A scatter plot of the estimated $\theta_{i^{\prime }}^{\left(1\right)}$ at the end of the second cycle and at the end of the fourth cycle is shown in Figure 2 (right). In patients 1^′^ to 3^′^, the points are near the diagonal, which suggests that $\theta_{i^{\prime }}^{\left(1\right)}$ of patients 1^′^ to 3^′^ are stable. In contrast, because the points of patient *i* ^′^ = 4^′^ are far from the diagonal, $\theta_{4^{\prime }}^{\left(1\right)}$ may remain unstable due to the insufficient AE data.

We assumed that the estimated $\theta_{i^{\prime }}^{\left(1\right)}$ was close enough to the true value at the end of the second cycle. We then calculated $\lambda_{1^{\prime }j}^{\left(1\right)}$ according to Equations (2) and (3) and listed the AE types most likely to occur in the future (Table 3). Those AE types should be carefully monitored.

**TABLE 3:** Top 10 AE types and top 10 severe AE types with the largest $\lambda_{1^{\prime }j}^{\left(1\right)}$

AE type	$\lambda_{1^{\prime }j}^{\left(1\right)}$
NAUSEA_2	1.02
DYSPEPSIA_1	0.61
NAIL DIS_1	0.55
CONSTIP_1	0.53
TASTE PERVERS_1	0.33
ASTHENIA_1	0.32
VASODILAT_2	0.31
DIARRHEA_1	0.29
HEADACHE_2	0.26
INSOMNIA_1	0.25
MENS DIS_3	0.091
INFECT_3	0.046
FEVER_3	0.013
ASTHENIA_3	0.012
HEADACHE_3	0.008
ANOREXIA_3	0.008
LEUKOPENIA_3	0.008
STOMATITIS_3	0.007
LEUKOPENIA_4	0.006
DIARRHEA_3	0.006

## 4 DISCUSSION

Our statistical model with patient‐level AE data extracted AE patterns specific for each treatment. We clarified that our method allowed interpretable patient‐level safety profiling in a clinical study such as the illustration of the data analysis in this paper. Our statistical model can use the number of occurrences of each AE in a patient to predict future AEs if the total number of occurrences is sufficiently large so that the estimated ***θ*** is close enough to the true value. The proposed method is also widely applicable to data obtained from clinical studies with patient‐level AEs, regardless of disease or treatment. We believe that this paper is of great significance to the current situation where patient‐level data is being released.

In Section 2.1, we excluded alopecia due to its common occurrence in patients. Such a frequent AE is called “stopwords” in the field of natural language processing (e.g., prepositions). It is common to remove such terms before matrix decomposition.^18^ If alopecia were included, many patterns would have contained alopecia, which would prevent pattern extraction and pattern interpretation. Therefore, we decided to exclude alopecia from the analysis in this paper.

A limitation that was noted in our method is that *K* must be determined before analysis (Section 2.2.3). For example, if we used *K* = 10, instead of pattern 6 and pattern 7, a mixture of those patterns would have been extracted. We used *K* = 15 after discussion because we thought they can be separated. Although nonparametric Bayesian methods can estimate *K*, further research is needed to examine whether it is effective for our model.

In our model, $\theta_{\mathrm{ik}}^{\left(t\right)}$ represents the responsiveness of patient *i* to treatment *t*. If $\theta_{:k}^{\left(t\right)}$ and $\theta_{:k}^{\left(t\right)}$ are strongly correlated, a patient may be advised to opt for alternate treatments wherein severe AEs seldom occur based on the correlation. In this paper, however, there was no strong correlation between treatments. For this reason, it was difficult to predict AEs caused by a certain treatment based on the AEs induced by a different one.

For the sake of simplicity, we assumed that (1) the dosages of each drug were the same across clinical studies, (2) treatments were not changed within a cycle, and (3) the order in which the AEs occurred was insignificant. Regarding (1), in practice, however, the dosages varied depending on the clinical studies. Regarding (2), treatments may change within a cycle due to drug withdrawal or dose reduction. (1) and (2) can be resolved by incorporating a dose–response model whose shape parameters are adjusted by the dosages actually administered. Regarding (3), the number, order, and timing of occurrences may all be important. This obstacle may be overcome by modeling the occurrence data as time series without summarizing them for each treatment period.

In this paper, we did not show the result of using patient information such as age or weight, however, these factors could be incorporated into our model. Because $\theta_{\mathrm{ik}}^{\left(t\right)}$ represents the responsiveness of patient *i* to treatment *t*, $\theta_{\mathrm{ik}}^{\left(t\right)}$ could be expressed using patient information as follows:

$$\begin{array}{ll} \mu_{\mathrm{ik}} & =f\left(x_{i}\right)\\ \theta_{\mathrm{ik}}^{\left(t\right)} & ∼\text{LogNormal}\left(\mu_{\mathrm{ik}},\sigma^{2}\right) \end{array}$$

where **x**_*i*_ is a vector of patient information for patient *i*. *μ*_*ik*_ is determined by a certain function *f* of **x**, and $\theta_{\mathrm{ik}}^{\left(t\right)}$ follows a lognormal distribution with *μ*_*ik*_ as a parameter. We can also incorporate exposure and other pharmacokinetics data into the model. In our study, we attempted to use age, weight, and laboratory measurements as **x**_*i*_ for the data. The result showed that these information were not useful predictors for $\theta_{:k}^{\left(t\right)}$ in this data. In future research, the effective integration of patient information into the model will be investigated.

Performance evaluation using artificial data is impractical due to the complexity of the AE data structure. Our method should be validated by applying it to larger datasets to use in a clinical study.

## CONFLICT OF INTEREST

The authors declare no conflicts of interest.

## Supporting information

**Table S1.** The estimated ***ϕ*** ^(*t*)^ for all treatments.

**Data S1.** AE data examples.

## Data Availability

The original data cannot be shared due to the rules of Project Data Sphere. The data on results and analysis methods are available in the supplementary material section and GitHub.^15^

## APPENDIX A.

**Case 1: one mechanism, multiple treatments, multiple studies, and multiple periods**

The superscript *m* in ***M*** ^(*m*)^ is removed. The log‐probability Equation (15) is reduced to:

$$\begin{array}{ll} & \log\;P\left({\tilde{\theta }}^{\left(t\right)},M,R^{\left(t\right)},{\tilde{\xi }}^{\left(s\right)},S^{\left(s\right)}\;|\;Y_{p},\sigma_{M},\sigma_{R},\sigma_{S},\sigma_{G}\right)\\ & =\sum_{p=1}^{P}\sum_{i=1}^{N_{p}}\sum_{j=1}^{J}\log\;\text{Poisson}\left(Y_{\mathrm{ij}}^{\left(p\right)}\;|\;C_{i}^{\left(p\right)}{\left[\exp{\tilde{\theta }}^{\left(t_{p}\right)}\text{softmax}\left(M+R^{\left(t_{p}\right)}\right)+\exp{\tilde{\xi }}^{\left(s_{p}\right)}\text{softmax}\left(S^{\left(s_{p}\right)}\right)\right]}_{\mathrm{ij}}\right)\\ & +\sum_{k=1}^{K}\left[\sum_{j\;\in \;J}\log\;N\left(M_{\mathrm{kj}}\;|\;0,\sigma_{M}^{2}\right)+\sum_{\left(j,j^{\prime }\right)\;\in \;J}\log\;N\left(M_{kj^{\prime }}\;|\;M_{\mathrm{kj}},\sigma_{G}^{2}\right)\right]\\ & +\sum_{t=1}^{T}\sum_{k=1}^{K}\left[\sum_{j\;\in \;J}\log\;N\left(R_{\mathrm{kj}}^{\left(t\right)}\;|\;0,\sigma_{R}^{2}\right)+\sum_{\left(j,j^{\prime }\right)\in J}\log\;N\left(R_{kj^{\prime }}^{\left(t\right)}\;|\;R_{\mathrm{kj}}^{\left(t\right)},\sigma_{G}^{2}\right)\right]\\ & +\sum_{s=1}^{S}\sum_{l=1}^{L}\left[\sum_{j\;\in \;J}\log\;N\left(S_{\mathrm{lj}}^{\left(s\right)}\;|\;0,\sigma_{S}^{2}\right)+\sum_{\left(j,j^{\prime }\right)\;\in \;J}\log\;N\left(S_{lj^{\prime }}^{\left(s\right)}\;|\;S_{\mathrm{lj}}^{\left(s\right)},\sigma_{G}^{2}\right)\right] \end{array}$$

**Case 2: one mechanism, one treatment, multiple studies, and multiple periods**

In addition to the above case 1, the superscripts *t* in ***β*** ^(*t*)^, ***θ*** ^(*t*)^, and ***ϕ*** ^(*t*)^ are removed. ***R*** ^(*t*)^ and Equations (10) and (13) are eliminated. Equation (6) is simplified to: $\Phi_{k}^{\left(t\right)}=\text{softmax}\left(M_{k}\right)$. The log‐probability Equation (15) is reduced to:

$$\begin{array}{ll} & \log\;P\left(\tilde{\theta },M,{\tilde{\xi }}^{\left(s\right)},S^{\left(s\right)}\;|\;Y_{p},\sigma_{M},\sigma_{S},\sigma_{G}\right)\\ & =\sum_{p=1}^{P}\sum_{i=1}^{N_{p}}\sum_{j=1}^{J}\log\;\text{Poisson}\left(Y_{\mathrm{ij}}^{\left(p\right)}\;|\;C_{i}^{\left(p\right)}{\left[\exp\tilde{\theta }\;\text{softmax}\left(M\right)+\exp{\tilde{\xi }}^{\left(s_{p}\right)}\text{softmax}\left(S^{\left(s_{p}\right)}\right)\right]}_{\mathrm{ij}}\right)\\ & +\sum_{k=1}^{K}\left[\sum_{j\;\in \;J}\log\;N\left(M_{\mathrm{kj}}\;|\;0,\sigma_{M}^{2}\right)+\sum_{\left(j,j^{\prime }\right)\;\in \;J}\log\;N\left(M_{kj^{\prime }}\;|\;M_{\mathrm{kj}},\sigma_{G}^{2}\right)\right]\\ & +\sum_{s=1}^{S}\sum_{l=1}^{L}\left[\sum_{j\;\in \;J}\log\;N\left(S_{\mathrm{lj}}^{\left(s\right)}\;|\;0,\sigma_{S}^{2}\right)+\sum_{\left(j,j^{\prime }\right)\;\in \;J}\log\;N\left(S_{lj^{\prime }}^{\left(s\right)}\;|\;S_{\mathrm{lj}}^{\left(s\right)},\sigma_{G}^{2}\right)\right] \end{array}$$

**Case 3: one mechanism, one treatment, one study, and multiple periods**

In addition to the above case 2, the superscripts *s* in ***α*** ^(*s*)^, ***ξ*** ^(*s*)^, and ***η*** ^(*s*)^ are removed. The log‐probability Equation (15) is reduced to:

$$\begin{array}{ll} \log\;P\left(\tilde{\theta },M,\tilde{\xi },S\;|\;Y_{p},\sigma_{M},\sigma_{S},\sigma_{G}\right) & =\sum_{p=1}^{P}\sum_{i=1}^{N_{p}}\sum_{j=1}^{J}\log\;\text{Poisson}\left(Y_{\mathrm{ij}}^{\left(p\right)}\;|\;C_{i}^{\left(p\right)}{\left[\exp\tilde{\theta }\;\text{softmax}\left(M\right)+\exp\tilde{\xi }\;\text{softmax}\left(S\right)\right]}_{\mathrm{ij}}\right)\\ & +\sum_{k=1}^{K}\left[\sum_{j\;\in \;J}\log\;N\left(M_{\mathrm{kj}}\;|\;0,\sigma_{M}^{2}\right)+\sum_{\left(j,j^{\prime }\right)\;\in \;J}\log\;N\left(M_{kj^{\prime }}\;|\;M_{\mathrm{kj}},\sigma_{G}^{2}\right)\right]\\ & +\sum_{l=1}^{L}\left[\sum_{j\;\in \;J}\log N\left(S_{\mathrm{lj}}\;|\;0,\sigma_{S}^{2}\right)+\sum_{\left(j,j^{\prime }\right)\;\in \;J}\log N\left(S_{lj^{\prime }}\;|\;S_{\mathrm{lj}},\sigma_{G}^{2}\right)\right] \end{array}$$

**Case 4: one mechanism, one treatment, one study, and one period (no baseline period)**

In addition to the above case 3, ***α*** ^(*s*)^, ***ξ*** ^(*s*)^, ***η*** ^(*s*)^, and **S** ^(*s*)^ are eliminated, and Equations (4), (7), (8), (11), and (14) are eliminated. The subscripts and superscripts *p* are removed. Equation (2) is simplified to *λ*_*ij*_ = *β*_*ij*_, and the log‐probability Equation (15) is reduced to:

$$\begin{array}{ll} \log\;P\left(\tilde{\theta },M\;|\;Y,\sigma_{M},\sigma_{G}\right) & =\sum_{i=1}^{N}\sum_{j=1}^{J}\log\;\text{Poisson}\left(Y_{\mathrm{ij}}\;|\;C_{i}{\left[\exp\tilde{\theta }\text{softmax}\left(M\right)\right]}_{\mathrm{ij}}\right)\\ & +\sum_{k=1}^{K}\left[\sum_{j\;\in \;J}\log\;N\left(M_{\mathrm{kj}}\;|\;0,\sigma_{M}^{2}\right)+\sum_{\left(j,j^{\prime }\right)\;\in \;J}\log N\left(M_{kj^{\prime }}\;|\;M_{\mathrm{kj}},\sigma_{G}^{2}\right)\right] \end{array}$$
